# COVID-19 versus applied infection control policies in a Major Transplant Center in Iran

**DOI:** 10.1186/s12962-023-00427-x

**Published:** 2023-02-27

**Authors:** Mojtaba Shafiekhani, Tahmoores Niknam, Seyed Ahmad Tara, Parviz Mardani, Khatereh Mirzad Jahromi, Sedigheh Jafarian, Sara Arabsheybani, Halimeh Negahban, Majid Hamzehnejadi, Zahra Zare, Khadijeh Ghaedi Ghalini, Ali Ghasemnezhad, Mahmoud Akbari, Reza Shahriarirad, Seyed Ali MalekHosseini

**Affiliations:** 1 Shiraz Transplant Research Center, Shiraz University of Medical Sciences, Shiraz, Iran; 2 Shiraz Transplant Center, Abu-Ali Sina Hospital, Shiraz University of Medical Sciences, Shiraz, Iran; 3 Department of Clinical Pharmacy, Faculty of Pharmacy, Shiraz University of Medical Sciences, Shiraz, Iran; 4 Department of Biostatistics, School of Medicine, Shiraz University of Medical Sciences, Shiraz, Iran; 5 Thoracic and Vascular Surgery Research Center, Shiraz University of Medical Sciences, Shiraz, Iran; 6 Department of Surgery, Shiraz University of Medical Sciences, Shiraz, Iran; 7 Student Research Committee, Shiraz University of Medical Sciences, Shiraz, Iran

**Keywords:** COVID-19, Coronavirus Disease 2019, Transplant, Iran, Kidney transplantation, Health Care Policy

## Abstract

**Background:**

Since Shiraz Transplant Center is one of the major transplant centers in Iran and the Middle East, this study was conducted to evaluate outcomes of the applied policies on COVID-19 detection and management.

**Methods:**

During 4 months from March to June 2020, patient's data diagnosed with the impression of COVID-19 were extracted and evaluated based on demographic and clinical features, along with the length of hospital stay and expenses.

**Results:**

Our data demonstrated that a total of 190 individuals, with a median age of 58, were diagnosed with COVID-19 during the mentioned period. Among these, 21 patients had a positive PCR test and 56 patients had clinical symptoms in favor of COVID-19. Also, 113 (59%) patients were classified as mild based on clinical evidence and were treated on an outpatient basis. Furthermore, 81 out of 450 cases (18%) of the healthcare workers at our center had either PCR of clinical features in favor of COVID-19. The mortality rate of our study was 11% and diabetes mellitus, hypertension were considered risk factors for obtaining COVID-19 infection. The direct cost of treatment and management of patients with COVID-19 amounted to 2,067,730,919 IRR, which considering the 77 patients admitted to Gary Zone per capita direct cost of treatment each patient was 26,853,648 IRR.

**Conclusion:**

We demonstrated that the COVID-19 pandemic had a noticeable influence on our transplant center in aspects of delaying surgery and increased hospital costs and burden. However, by implanting proper protocols, we were able to was able to provide early detection for COVID-19 and apply necessary treatment and prevention protocols to safeguard the patients under its coverage, especially immunocompromised patients.

## Introduction

In late December 2019, China reported an outbreak of viral pneumonia in Wuhan, Hubei Province, China, which spread rapidly to other areas [1, 2]. The novel coronavirus disease 2019 (Also known as SARS-CoV-2 or COVID-19) is a global concern and has become a significant health problem since the number of infected cases and affected countries has escalated rapidly [3]. The outbreak of coronavirus in Iran was officially confirmed on February 19, 2020 [4]. On March 11, 2020, the World Health Organization (WHO) confirmed COVID-19 a pandemic. As of August 29, 2020, over twenty-five million cases of COVID have been reported with a death toll of around 843,000 patients and only around seventeen million recovered cases in 213 countries and territories worldwide. Among the top-ranking countries, Iran has placed in the twelfth position with over 371,000 confirmed cases and over 21,000 deaths [5]. Although months into the pandemic, there are still various aspects of the virus which remain unknown resulting in challenges in detecting and treating the disease; therefore, many medical centers with large numbers of patients are requiring special care settings and subsequently impose high work pressure on medical staff and increase medical costs causing a significant burden on health care systems.

Meanwhile, patients with special clinical conditions such as receiving a transplanted organ or waiting for a transplant due to the weakened condition of their immune system are more prone to infection and its complications [6, 7], therefore, centers providing service and care for these patients must adopt appropriate and consistent a strategy [8]. Recent case series from the UK and Italy have reported a mortality of 7–25% of COVID-19 in post-transplant patients, adding to the concern that these patients suffer significantly adverse outcomes [9]. Akdur and associates reported that the disease course for kidney transplant recipients infected with SAR-CoV-2 was worse than shown in the normal population. Compared with the overall mortality rate in the United States of 1% to 5% (and up to 15% in patients over 70 years of age), the mortality rate in their centers was 28% in solid-organ transplant recipients [10].

In this study, we aim to study aspects of COVID-19 and experiences in this field in Shiraz Organ Transplant Hospital as one of the largest transplant centers in Asia from March to June 2020.

## Method

### Study design and data collection

This retrospective study was conducted in Shiraz Transplant Hospital, Shiraz, Fars, Iran affiliated by Shiraz University of Medical Sciences as one of the largest centers for solid organ transplantation with more than 500 liver transplantation and 300 kidney transplantations annually and more than 300 hospital beds and 22 active wards. The center also covers a variety of additional wards such as internal medicine, cardiology, pulmonology, gastrointestinal, and other surgical wards. The study timeline was during a 4-month period from March to June 2020 in which all patients diagnosed with COVID-19 were enrolled either based on SARS-CoV-2 positive real-time polymerase chain reaction (RT-PCR), or clinical manifestations along with radiographic findings with hospitalized for more than 48 h. Demographic information of patients along with the course of disease and array of signs and symptoms, medical history, clinical and radiographic findings, length of hospital stay, and clinical outcome of all patients were extracted from the medical records of the patients and evaluated accordingly.

### Screening and triage

With the establishment of a fever clinic at the entrance of the hospital, which consists of two nurses and a general practitioner on a 24-h basis, all staff, patients, and patient companions were first assessed by a digital thermometer on a daily basis. In terms of fever or suspicious clinical symptoms, the physician will then first perform a physical examination and obtain a persist medical and contact history, and in suspicious cases, referred to an infectious disease specialist residing in the hospital for further evaluation. RT-PCR was used to confirm suspected cases. RT-PCR assays performed following the protocol established by the WHO [11]. All suspected patients were initially evaluated by internal medicine and infectious disease specialists and based on the severity of clinical symptoms, radiographic images, and PCR test results were divided into three categories: mild, moderate, and severe [12], and if decided by internal medicine and infectious disease specialists, they were transferred to the quarantine ward for patients with COVID-19 (The Gray-Zone). All patients were evaluated daily by transplant surgeons, internal medicine, infectious, pulmonary, and pharmacotherapy specialists, and the necessary medical or surgical interventions were done for management and treatment.

### Transplantation and surgeries policies

With the widespread of this virus in the country and Fars province, according to the instructions issued by the Ministry of Health, all non-emergency surgeries were suspended and only emergency surgeries were performed, which in these cases in our hospital, candidates will undergo a full screening including obtaining medical and contact history, full physical examination, SARS-CoV-2 PCR, and if required radiographic evaluations by a general physician which is normally conducted only for surgical cases. In the case of transplant surgery, the donor and recipient of the solid organ must have a negative PCR test as well as the absence of any radiographic findings or suspicious symptoms in favor of COVID-19 during the past 48 h before transplant, which will be evaluated by a team of infectious disease specialists, transplant surgeons, and internal medicine specialists. Furthermore, donors who were among residents with a high prevalence of COVID-19 and considered as a “red areas” according to the announcement of the Ministry of Health were not eligible for organ donation in the initial two months. The center also set up a virtual clinic to monitor transplanted patients or waiting for solid transplant who weren’t admitted at our center, so that all patients are virtually visited daily by transplant surgeons and answered their inquiries if needed.

### Management and treatment of COVID-19

In mild patients which were dischargeable based on the infectious and internal medicine specialist's opinion and followed on an outpatient basis, instructions for receiving hydroxychloroquine and related health tips related to self-quarantine for 14 days were provided. They were also daily telephone monitoring by nurses specialized in infection control for the initial 10 days, and if the disease progressed were re-visited at our clinic. In moderate and severe patients, depending on the underlying disease, received treatment regimen consisting of hydroxychloroquine along with Lopinavir/Ritonavir based on the recommended protocol ordered by the Health Ministry of Iran at the time of detection of infection [13, 14].

In transplant recipients, the basis of pharmacotherapy was based on the principles mentioned by Mirjalili et al. [15]. The condition for the discharge of the patient or transfer from the “Grey Zone" to other wards was the improvement of clinical symptoms, the presence of two negative PCR tests with an interval of 48 h, and respiratory rate below 22 with oxygen saturation percentage (SPO_2_%) above 95% without the need for oxygen therapy or ventilation.

### Statistical analysis

Categorical variables were described as frequency rates and percentages, and continuous variables were described using mean, median, and interquartile range (IQR) values. Means for continuous variables were compared using independent group t-tests when the data were normally distributed; otherwise, the Mann–Whitney test was used. Data (non-normal distribution) from repeated measures were compared using the generalized linear mixed model. Proportions for categorical variables were compared using the χ^2^ test, although the Fisher exact test was used when the data were limited. All statistical analyzes were performed using SPSS (Statistical Package for the Social Sciences) version 26.0 software (SPSS Inc). For unadjusted comparisons, a 2-sided α of less than 0.05 was considered statistically significant.

## Results

During the study period, 190 patients suspected of having COVID-19 were diagnosed based on clinical signs, radiographic findings, and PCR results with a median age of 58 (IQR: 44–66.25). Among these, 21 patients had a positive PCR test for COVID-19 and 56 patients had clinical symptoms along with highly suspicious radiographic findings for COVID-19, who were admitted to the gray zone ward. Furthermore, 113 patients (59.47%) were classified as mild based on clinical evidence and were treated on an outpatient basis and 77 patients (40.52%) accounted for as moderate and severe. Table 1 shows the demographic and clinical information related to patients admitted to the Gray zone (N = 77) by patients with a positive and negative PCR test.

**Table 1:** Demographic and clinical features of admitted COVID-19 patients in Shiraz Transplant Center (N = 77)

Variable	COVID-19 PCR Positive (%) *n* = *21*	COVID-19 PCR negative patients but highly suspicious (%) *n* = *56*
Age group		
< 40	3 (14.3)	7 (10.7)
40–60	10 (47.6)	17 (28.6)
> 60	8 (38.1)	32 (57.1)
Gender		
Male	12 (57.1)	38 (67.9)
Female	9 (42.9)	18 (32.1)
Residence (Fars province)		
Shiraz (capital of Fars)	9 (42.9)	34 (60.7)
Other Cities	12 (57.1)	22 (39.2)
Comorbid diseases		
Diabetes mellitus	6 (28.5)	17 (30.4)
Cirrhosis	5 (23.8)	14 (25)
Hypertension	4 (19.0)	18 (32.1)
Ischemic heart disease	2 (9.5)	1 (1.8)
Encephalopathy	1 (9.5)	5 (8.9)
Asthma	1 (4.8)	0 (0)
Ascites	1 (4.8)	3 (5.4)
End-stage renal disease	1 (4.8)	7 (12.5)
Chronic obstructive pulmonary disease	0 (0)	4 (7.1)
Deep venous thrombosis	0 (0)	1 (1.8)
Symptoms		
Fever	11 (52.4)	17 (30.4)
Cough	6 (28.6)	13 (23.2)
Dyspnea	6 (28.6)	20 (35.7)
Vomiting	5 (23.8)	8 (14.3)
Diarrhea	3 (14.3)	6 (10.7)
Headache	3 (14.3)	2 (3.6)
Pharyngitis	0 (0)	0 (0)
Rhinorrhea	0 (0)	0 (0)
Chest pain	0 (0)	0 (0)
Malaise	0 (0)	0 (0)
Transplantation Status		
Liver transplant	5 (23.8)	4 (7.1)
Kidney transplant	3 (14.3)	4 (7.1)
Candidate for transplant	8 (38.1)	15 (26.8)
Time after transplant (months)		
1–3	0 (0)	2 (25)
3–6	2 (22.2)	2 (25)
6–12	3 (33.3)	1 (12.5)
> 12	4 (44.4)	3 (37.5)

*COVID-19* Coronavirus disease 2019, *PCR* Polymerase chain reaction.

The mean age of patients was 55.8 (± 16.2) for PCR positive and 54.28 (± 17.2) for the clinical suspicious group, with no significant correlation among the two groups (P = 0.70). The results of quantitative and qualitative factors as risk factors for COVID 19 based on regression analysis are shown that the presence of diabetes mellitus, hypertension, and transplantation were the three risk factors for COVID-19 in patients in our study.

Also, a mortality rate of 21 (11%) cases was reported in our center in which 16 (76.19%) had positive PCR for COVID-19.

As demonstrated in Table 1, 16 transplant patients (7 kidney transplant and 9 liver transplant patients) are seen in patients with positive and highly suspicious PCR tests, 44% of which have been transplanted more than 12 months ago and 23 patients were on the transplant waiting list.

According to the policies adopted in the hospital, which was mentioned in the method section, the number of patients admitted during the study period had decreased by 44% compared to the same period last year, and in this regard, the number of general surgeries performed had decreased by 20%, liver transplantations 50.5% and kidney transplantations by 22.48% compared to the same period last year. At the time of the study, approximately 33% of the hospital's medical and administrative staff (450 cases) had been screened at least once for COVID-19 disease by PCR testing in which 14 hospital staff (including 3 physicians, 8 nurses, and 3 staff members) had a positive PCR result and 67 cases had a clinical symptom suggestive of COVID-19 with negative PCR. Among these groups, 51 (65.43%) reported close contact with a patient with COVID-19 during their work process. It is also worth mentioning that all of the personnel reported having access to personal protective equipment (PPE).

Based on the results extracted from the hospital database until the end of this study, the direct cost of treatment and management of patients with COVID-19 (admitted in Gary Zone) amounted to 2,067,730,919 IRR (approximately 51,000 USD), which considering the 77 patients admitted to Gary Zone per capita direct cost of treatment each patient was 26,853,648 IRR. Furthermore, the cost of purchasing PPE and disinfectants during this period was 8,420,016,600 Rials.

## Discussion

With the ongoing COVID-19 pandemic, transplant patients and those with end-stage organ failure are in a particularly vulnerable position and are impacted by the disease from various aspects. Among the problems transplant patients endure one could name that elective surgeries such as live donor transplant procedures have been put on hold in many countries [16, 17]. Also, a decrease in the number of donor transplants, where the procedure is established, continues in some countries, albeit with modified donor and recipient criteria, in an attempt to reduce the risk of COVID-19 transmission or infection after transplantation [17, 18]. Additionally, administered immunosuppression drugs in these patients increase the risk of disease contraction and progression [6, 8]. With the current situation, adopted policies will result in a loss of transplant opportunities and therefore a substantial increase in the number of waiting list patients with also a significant impact on patient and hospitals, such as an increase in dialysis capacity and provision for kidney transplant patients, with additional inevitable morbidity and mortality; Hence, caring for patients who are at higher risk of disease progression is vital during this distinct period.

Our study demonstrated that 59.47%patients had a mild presentation of COVID-19. In a similar study, Pereia et al. [19] reported 90 patients in an epicenter in the United States, among these, twenty‐two (24%) had mild, 41 (46%) moderate, and 27 (30%) severe disease. The higher rate of mild patients in our study can be contributed to the implanted policies for screening and early detection of the disease, which subsequently resulted in a higher detection rate along with identifying patients at early stages of the disease, also some studies declared transplanted patients due to their immunosuppression status presented with less severe symptoms [20, 21]. However, relying on molecular detection of the disease rather than the clinical presentations for commencing treatment is still a controversial issue [22].

The mortality rate in our study was 11% which was higher than the reported rate of the province (8%) [23]. Also, Pacual et al. reported a fatality rate of about 45.8% during the first 60 days after kidney transplantation [24]. The mortality rate varies among studies which may be due to the patients included and comorbidities, which in our case the major cause of mortality was ARDS. So, special attention should be given to decelerate the progression of the disease and avoid reaching severe phases. Diabetes mellitus and hypertension were the most detected comorbidities in our study, which was also reported in other studies [19, 23], which has been reported to be linked with ACE2-increasing drug treatment in these patients [25].

Regarding personnel and healthcare worker infection rates, 81 out of 450 cases (18%) had either PCR or clinical features in favor of COVID-19. Based on PCR results, only 14 (3.1%) had positive PCR tests, which was lower than a previous study in our province which demonstrated an infection rate of 5.6% among healthcare workers [26]. Chu et al. [27] reported a rate of 57 cases during 5 weeks by the means of clinical presentations and based on WHO interim guidance [28], our study also demonstrated a higher detection rate by exploiting clinical presentations, in which 67 cases were detected by this method. Whether molecular or clinical features should be used in the context of detecting COVID-19 cases is still a matter of debate which reports highlight employing clinical features due to the high rate of PCR false negatives [22]. Furthermore, the highest group at risk of infection was nurses which accounted for 8 out of 14 (57.1%) positive PCRs for COVID-19 in our study versus a 51.3% rate in a previous study in Fars [26]. This fact may be due to that nurses have more patient contact in comparison with other healthcare workers which increases the risk of infection [29, 30]. Although safety measures and self-protection equipment were provided for all health care workers, these populations are still at risk and should be routinely screen to provide early detection and treatment.

This is while a person working in Iran typically earns around 44,800,000 IRR per month (11,300,000 IRR lowest average) [31], while based on our data, the average cost for COVID-19 treatment among the patients in our study was around 10,800,000 IRR, which is 23% to 95% of an average individual’s salary. Other studies have reported that a maximum cost of 20,000 USD for COVID-19 inpatient admissions [25]. This is aside from the substantial cost of purchasing PPE and disinfectants during this period imposed on the hospital along with the decreased number of elective surgeries which subsequently results in a significant reduction in the income for hospitals, which is mentioned in other studies [32, 33]. Therefore, these facts, along with other studies, demonstrate that the global health issue caused by the COVID-19 pandemic not only impacts the individual’s health, but also the economy, mental status, and other various aspects [25, 34–36], therefore considering all these factors is vital in going on with this global pandemic.

Although this study provides optimistic evidence of controlling the disease, some limitations remain. Our report was during the initial four months of the pandemic in Iran, and long-term follow-up of the discharged patients are not available. This is while the disease is still persistent in the country and many centers and individuals are still struggling with its impact, therefore precise evaluating management and therapeutic options is vital for optimizing the ultimate results.

## Conclusion

We demonstrated that the COVID-19 pandemic had a noticeable influence on our transplant center in aspects of delaying surgery and increased hospital costs and burden. We also demonstrated that with the applied policies in the Shiraz transplant center, effective detection, control, and management of the disease were obtained. Although COVID-19 incidence in transplant cases was relatively high, early detection and management are important factors in preventing the spread of the disease. Further reports on the long-term effect of these applied protocols, along with necessary and up-to-date modifications are necessary to provide the optimal control and management of the disease.

## Data Availability

All data generated or analyzed during this study are included in this manuscript. Please write to the corresponding author for further information.

## Abbreviations

COVID-19: Novel coronavirus disease 2019
IQR: Interquartile range
PPE: Personal protective equipment
RT-PCR: Real-time polymerase chain reaction
SPSS: Statistical Package for the Social Sciences
WHO: World Health Organization
